# Resistance of *Blastocystis* to chlorine and hydrogen peroxide

**DOI:** 10.1007/s00436-022-07713-2

**Published:** 2022-11-15

**Authors:** Rubén Martín-Escolano, Geok Choo Ng, Kevin S. W. Tan, C. Rune Stensvold, Eleni Gentekaki, Anastasios D. Tsaousis

**Affiliations:** 1 Laboratory of Molecular and Evolutionary Parasitology, RAPID Group, School of Biosciences, University of Kent, Canterbury, CT2 7NJ UK; 2 Department of Microbiology and Immunology, Yong Loo Lin School of Medicine, National University of Singapore, 5 Science Drive 2, Singapore, 117545 Singapore; 3 Department of Bacteria, Parasites and Fungi, Statens Serum Institut, Artillerivej 5, 2300 Copenhagen S, Denmark; 4 Gut Microbiome Research Group, Mae Fah Luang University, Chiang Rai, 57100 Thailand; 5 School of Science, Mae Fah Luang University, Chiang Rai, 57100 Thailand

**Keywords:** *Blastocystis*, Chlorine resistance, Hydrogen peroxide, Transmission dynamics, Water

## Abstract

*Blastocystis* is a ubiquitous, widely distributed protist inhabiting the gastrointestinal tract of humans and other animals. The organism is genetically diverse, and so far, at least 28 subtypes (STs) have been identified with ST1–ST9 being the most common in humans. The pathogenicity of *Blastocystis* is controversial. Several routes of transmission have been proposed including fecal–oral (e.g., zoonotic, anthroponotic) and waterborne. Research on the latter has gained traction in the last few years with the organism having been identified in various bodies of water, tap water, and rainwater collection containers including water that has been previously filtered and/or chlorinated. Herein, we assessed the resistance of 11 strains maintained in culture, spanning ST1–ST9 to various chlorine and hydrogen peroxide concentrations for 24 h, and performed recovery assays along with re-exposure. Following the treatment with both compounds, all subtypes showed increased resistance, and viability could be visualized at the cellular level. These results are hinting at the presence of mechanism of resistance to both chlorine and hydrogen peroxide. As such, this pilot study can be the platform for developing guidelines for water treatment processes.

**Supplementary Information:**

The online version contains supplementary material available at 10.1007/s00436-022-07713-2.

## Introduction

*Blastocystis* is one of the most commonly encountered microbial eukaryotes in the gastrointestinal tract of humans and a wide range of other animals (Alfellani et al. 2013b; Tsaousis et al. 2020). The organism is distributed globally having been identified in both developed and developing countries in rural and urban settings (Scanlan et al. 2014; Udonsom et al. 2018).

*Blastocystis* exhibits remarkable genetic diversity, and at least 28 subtypes (STs, ST1-ST17, ST21, ST23–32) – arguably species – have been identified in humans, other mammals, and birds, based on genetic heterogeneity across the small subunit rRNA (*SSU* rRNA) gene (Maloney et al. 2020; Stensvold and Clark 2020; Higuera et al. 2021). Of these subtypes, ST1–ST9, ST10, ST12, ST14, ST16, and ST23 have been found in humans, with ST1–ST3 being the three most prevalent and globally distributed (Yoshikawa et al. 2004; Meloni et al. 2011; Forsell et al. 2012; Khaled et al. 2020; Jinatham et al. 2021; Osorio-pulgarin et al. 2021). However, these subtypes have also been found in several other hosts, indicating the lack of host specificity of *Blastocystis* (Stensvold and Clark 2016), at least at subtype level. The exception appears to be ST9, which has so far been exclusively isolated from humans. Zoonotic transmission of the organism has been suggested (Abe et al. 2003; Stensvold et al. 2009).

It has been speculated that *Blastocystis* can remain in the intestine for weeks, months, or even years though this has yet to be conclusively demonstrated (Scanlan et al. 2014). Nonetheless, its pathogenicity remains unclear. *Blastocystis* infection has been linked to gastrointestinal symptoms, the main ones being watery or loose stools, diarrhea, excessive gas, abdominal pain, anal itching, and weight loss (Booroom et al. 2008; Stensvold et al. 2011). Links to irritable bowel syndrome and inflammatory bowel disease have also been postulated though not conclusively established (Domínguez-márquez et al. 2009; Roberts et al. 2013; Salvador et al. 2016; Peña et al. 2020; Shirvani et al. 2020). However, *Blastocystis* is also very common in the gut of people with no gastrointestinal symptoms (Nagel et al. 2012; Scanlan et al. 2015; Yowang et al. 2018; Jinatham et al. 2021). Hence, it is possible that *Blastocystis* colonization in general is not harmful, but rather specific subtypes or strains within subtypes might be the ones potentially causing symptomology.

Although the transmission dynamics of *Blastocystis* remain blurry, it is widely understood that the organism enters the host via the fecal–oral route (Tan 2004). The precise contribution of the various forms (i.e., cyst, granular, vacuolar and amoeboid) of the organism to transmission and colonization/infection is unknown. Several factors have been linked with increased occurrence of *Blastocystis* with waterborne transmission featuring prominently (Anuar et al. 2013; Deng et al. 2020; Salazar-Sanchez et al. 2021). *Blastocystis* has been detected in drinking water (Leelayoova et al. 2008), tap water (Eroglu and Koltas 2010; Jinatham et al. 2022), rainwater tanks (Waters et al. 2019; Jinatham et al. 2021), bodies of freshwater (Khalifa et al. 2014), drinking water treatment facilities (Richard et al. 2016; Freudenthal et al. 2022), and wastewater (Stensvold et al. 2020) worldwide.

Chlorine is one of the most widely used reagents for disinfection of water. A single study showed the potential of *Blastocystis* to resist chlorine; however, this study preceded the implementation of the subtyping system (Zaki et al. 1996). Hence, it is unknown whether chlorine resistance might be subtype- or strain-specific. The longevity of the organism in the environment and how it deals with oxidative stress has also been subject to investigation. Previous studies have shown that *Blastocystis* has mechanisms to withstand oxidative stress; however, these were based on in silico predictions or were performed experimentally in a limited number of strains (Tsaousis et al. 2012; Eme et al. 2017; Gentekaki et al. 2017). In this pilot study, a resazurin-based assay was used to test the resistance of eleven *Blastocystis* isolates representing ST1 through ST9 to chlorine and hydrogen peroxide.

## Materials and methods

### *Blastocystis* spp. isolates

Eleven different *Blastocystis* isolates from nine subtypes (Table 1) were used to test resistance to chlorine and hydrogen peroxide. Both xenic and axenic cultures were used. Xenic refers to mono-eukaryotic (containing only *Blastocystis*) cultures with bacteria, while axenic refers to cultures that only contain *Blastocystis*.

**Table 1:** *Blastocystis* isolates and subtypes used to test resistance to chlorine and hydrogen peroxide

Isolate	Subtype	Culture	Source	Country	Reference
NUH9	1	Axenic	Human	Singapore	Wong, Kenneth H.S. et al. 2008
HJ96-1	2	Xenic	Human	Japan	Yoshikawa, H. et al.^ 2003^
HJ96A-26	3	Xenic	Human	Japan	Yoshikawa, H. et al. 2000
S1	4	Xenic	Rodents	Singapore	Tan, 2008
WR1	4	Axenic	Rodents	Singapore	Chen, X.Q. et al. 1997
SY94-3	5	Xenic	Pig^a^	Japan	Yoshikawa, H. et al. 1998
HJ96AS-1	6	Xenic	Human	Japan	Yoshikawa, H. et al. 2000
H	7	Axenic	Human	Singapore	Ho, L.C. et al. 1994
B	7	Axenic	Human	Singapore	Ho, L.C. et al. 1994
MJ99-132	8	Xenic	Primate^b^	Japan	Abe, N. et al. 2003
HJ00-4	9	Xenic	Human	Japan	Yoshikawa, H. et al. 2004

^a^*Sus scrofa*; ^b^*Varecia variegate*

### *Blastocystis* spp. cell culturing

*Blastocystis* isolates were cultured in an anaerobic chamber at 37 °C in Iscove’s Modified Dulbecco’s Media (IMDM) (Gibco) supplemented with 10% (v/v) heat-inactivated horse serum (hiHS) (Thermo Fisher Scientific). Cultures were maintained in sterile 14-mL round-bottom polystyrene tubes (Thermo Scientific) in a GasPak™ EZ Anaerobe Container System (GasPak™ jar crystal with GasPak™ Anaerobe sachets) (Ho et al. 1993; Clark and Diamond 2002).

Cells were maintained by passages – 1-mL gently homogenized culture to 9-mL fresh medium – every 4 to 7 days, depending on their growth. Fresh medium was de-gassed and warmed to 37 °C a minimum of 48 h before the cultures were passaged. Cultures were routinely evaluated using light microscopy for growth, morphology, and contaminants. For the assays described below, cultures at the logarithmic phase were used (primarily vacuolar and secondarily granular forms).

### Exposure to chlorine and hydrogen peroxide and resazurin-based viability assays

Resistance of *Blastocystis* to chlorine and hydrogen peroxide was assessed using 96-well flat-bottom microtiter plates by seeding 5 × 10^5^ *Blastocystis* cells/well and after addition of the reagents to be tested in 200 µL/well volumes in IMDM supplemented with 10% (v/v) hiHS under anaerobic conditions at 37 °C. Cell concentration was determined quantitatively by the trypan blue dye exclusion method (Roberts et al. 2015; Mokhtar et al. 2019), using an automatic cell counter (EVE, NanoEntek). Chlorine and hydrogen peroxide were serially diluted to reach final concentrations ranging from 5000 to 2 mg/L (ppm) and from 10 to 0.001% (w/w) in plates, respectively. The source of chlorine was a sodium hypochlorite (NaoCl) solution containing 10% of the elemental compound. Blanks (containing only phosphate-buffered saline [PBS]), negative (containing only culture medium), and positive (untreated cells) growth controls were also included. A 30% commercially available hydrogen peroxide solution was used (ACROS organics). After 24 h of incubation, 20 µL of a 0.125-mg/mL resazurin sodium salt solution (Sigma-Aldrich) was added into each well with subsequent anaerobic incubation for further 3–5 h at 37 °C (Mirza et al. 2011; Yason et al. 2018). Finally, 20 µL of 20% (w/v) sodium dodecyl sulfate (SDS) was added, and after 20 min, cell viability was assessed by fluorescence measurements at 544/590 nm (ex/em) wavelengths using a FLUOstar® Omega microplate reader.

Relative fluorescence units (RFU) were converted into viability percentages: negative control values, which are taken as 0% growth, were subtracted from the rest of the fluorescence values; later, viability percentages were calculated with respect to positive controls, which are taken as 100% growth. These viability percentages were used to perform nonlinear regression analyses using GraphPad Prism 6 to determine the IC_50_, IC_90_, and IC_99_ values, i.e., the concentrations required to result in 50%, 90%, and 99% growth inhibition. Experimental minimum inhibitory concentrations (MICs) were also determined. Each reagent concentration was tested in triplicate in three separate determinations.

### Recovery assays

Recovery of *Blastocystis* to chlorine and hydrogen peroxide was assessed using 96-well flat-bottom microtiter plates by seeding 5 × 10^5^ *Blastocystis* cells/well after addition of the reagents to be tested in 200-µL/well volumes in IMDM supplemented with 10% (v/v) hiHS under anaerobic conditions at 37 °C. Chlorine and hydrogen peroxide were serially prepared as described above. Blanks, negative, and positive (untreated) growth controls were also included.

After a 24-h incubation, plates were centrifuged at 1,200 × *g* for 5 min and carefully washed three times with 200-μL/well volume pre-warmed IMDM, followed by a 24-h incubation without reagent treatments in IMDM supplemented with 10% (v/v) hiHS under anaerobic conditions at 37 °C. Finally, cell viability was determined by fluorescence measurements as described above (Mirza et al. 2011; Yason et al. 2018). IC_50_, IC_90_, and IC_99_ values were determined, as well as experimental minimum lethal concentrations (MLCs) (Roberts et al. 2015). Each reagent concentration was tested in triplicate in three separate determinations.

### Fluorescence live-cell imaging

To provide representative images of *Blastocystis*, random microscopic fields were captured from untreated and treated cultures of *Blastocystis* S1 (ST4, xenic), WR1 (ST4, axenic), H (ST7, axenic), and B (ST7, axenic). In short, *Blastocystis* STs were seeded at 1 × 10^6^ cells/well in 12-well plates after the addition of the reagents at the IC_50_ final concentrations in 2-mL volumes in IMDM supplemented with 10% (v/v) hiHS under anaerobic conditions at 37 °C. Untreated cultures were also included. After a 24-h incubation, cells were centrifuged at 800 × *g* for 10 min, carefully washed three times with PBS, and resuspended in PBS containing 200-nM MitoTracker™ Red CMXRos, a mitochondrion-specific stain that has been used previously on *Blastocystis* (Stensvold et al. 2007; Tsaousis et al. 2012). Finally, *Blastocystis* cells were incubated anaerobically for 40 min in the dark, and images were taken through bright and red filters using the JuLI™ Stage System for live-cell imaging. The same software was used to automatically count the fluorescent cells versus the total number of cells.

## Results

### Chlorine resistance assays

**Fig. 1:**
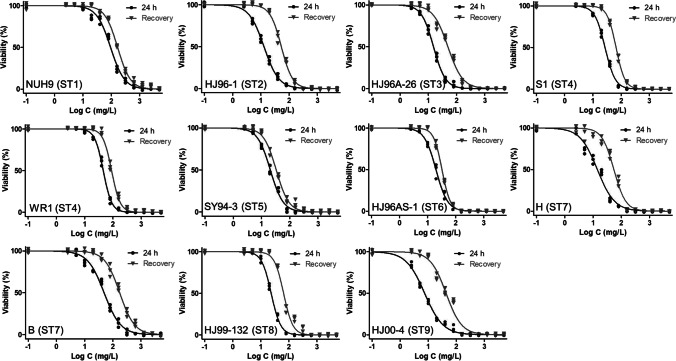
Dose–response curves for each *Blastocystis* isolate against chlorine using GraphPad Prism 5 software. Each reagent concentration was tested in triplicate in three separate determinations (averaged)

Figure 1 shows the dose–response curves, and Table 2 summarizes the IC and MIC values for each *Blastocystis* isolate against chlorine after 24 h of treatment and recovery. After 24 h of treatment, all isolates showed IC_50_ concentrations (≥ 7.4 ppm) higher than the chlorine concentrations used to disinfect water (up to 5 ppm) (Zaki et al. 1996; Yang et al. 2018; Centers for Disease Control and Prevention 2020; Karim et al. 2020). With regard to disinfection, the IC_99_ concentrations are the relevant ones, with values considerably higher (≥ 140 ppm) for all the isolates tested. When MIC concentrations are considered, these values increased to higher than 300-ppm chlorine after 24 h of treatment. Notably, ST8 showed the highest sensitivity to chlorine, with an IC_99_ value of 140.3 ppm. In contrast, ST1 showed the highest resistance to chlorine, showing an IC_99_ value of 1,268 ppm, followed by ST7 strain B at 1,079 ppm.

**Table 2:** Activity of chlorine (ppm) against the *Blastocystis* isolates

24-h treatment					
Isolate	Subtype	IC_50_ (ppm)	IC_90_ (ppm)	IC_99_ (ppm)	MIC (ppm)
NUH9	1	94.5	327.1	1268.0	2500.0
HJ96-1	2	12.9	48.8	208.7	312.5
HJ96A-26	3	15.4	44.9	145.3	156.3
S1	4	26.9	67.4	183.6	312.5
WR1	4	45.4	96.8	221.0	312.5
SY94-3	5	20.6	96.3	228.6	312.5
HJ96AS-1	6	19.8	81.1	156.8	312.5
H	7	14.2	66.4	356.6	625.0
B	7	49.1	215.2	1079.0	1250.0
MJ99-132	8	23.3	55.0	140.3	156.3
HJ00-4	9	7.4	39.4	243.6	312.5
24-h recovery					
Isolate	Subtype	IC_50_ (ppm)	IC_90_ (ppm)	IC_99_ (ppm)	MIC (ppm)
NUH9	1	167.9	524.4	1817.0	5000.0
HJ96-1	2	53.1	145.9	439.7	625.0
HJ96A-26	3	49.4	189.0	791.7	1250.0
S1	4	66.1	146.0	346.5	625.0
WR1	4	89.7	192.8	444.6	625.0
SY94-3	5	33.3	151.4	353.7	625.0
HJ96AS-1	6	32.5	89.1	178.6	312.5
H	7	54.6	158.1	504.6	625.0
B	7	175.5	666.2	2857.0	5000.0
MJ99-132	8	66.8	169.7	469.9	625.0
HJ00-4	9	43.1	177.7	833.4	1250.0

IC, inhibitory concentration; MIC, minimum inhibitory concentration. Axenic cultures in blue; xenic cultures in red

Recovery assays were performed to determine the static or cidal activity of chlorine against *Blastocystis*. All isolates showed recovery after 24 h of incubation without chlorine treatment (Fig. 1), suggesting that resistance forms (cysts) are developed during treatment and subsequently allow *Blastocystis* recovery. Concentrations ranging from 178 to higher than 2,857 ppm were required to completely eliminate any chance of recovery (Table 2, 24-h recovery) of the studied strains. Similar to the treatment assays, ST1 and ST7 showed the highest resistance to chlorine with IC99 at 1,817 ppm and 2,857 ppm, respectively.

### Hydrogen peroxide resistance assays

**Fig. 2:**
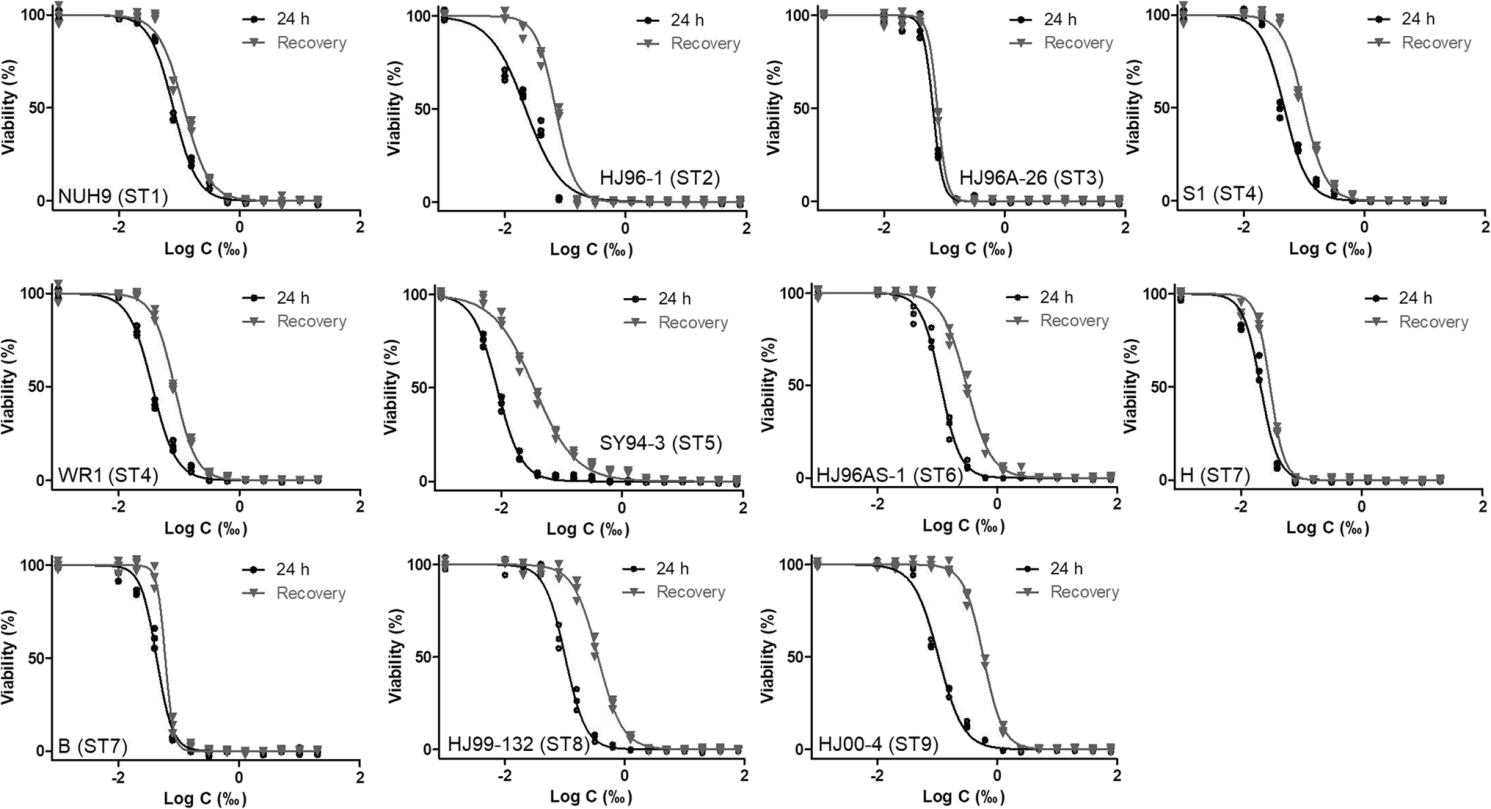
Dose–response curves for each *Blastocystis* isolate against hydrogen peroxide using GraphPad Prism 5 software. Each reagent concentration was tested in triplicate in three separate determinations (averaged)

Figure 2 shows the dose–response curves, and Table 3 summarizes the IC and MLC values for each *Blastocystis* isolate against hydrogen peroxide after 24 h of treatment and recovery. All isolates exhibited IC_50_ concentrations ranging from 8.5 ppm to 113.8 ppm after 24 h of treatment and IC_99_ disinfectant concentrations ranging from 72.8 to 946.6 ppm. The MLC concentrations ranged from 156 to 1250 ppm. Of note, ST5 showed the highest sensitivity to hydrogen peroxide, with an IC_99_ of 72.8 ppm. In contrast, ST9 was the strain that was most resistant to hydrogen peroxide, showing an IC_99_ of 946.6 ppm, followed by ST6 at 650.9 ppm and ST1 at 641.9 ppm.

**Table 3:** Activity of hydrogen peroxide (ppm) against the *Blastocystis* isolates

24-h treatment					
Isolate	Subtype	IC_50_ (ppm)	IC_90_ (pmm)	IC_99_ (ppm)	MLC (ppm)
NUH9	1	79.6	216.0	641.9	1250.0
HJ96-1	2	22.2	163.5	380.2	625.0
HJ96A-26	3	65.0	113.0	154.2	312.5
S1	4	46.9	125.4	367.1	625.0
WR1	4	36.1	97.3	287.8	312.5
SY94-3	5	8.5	33.7	72.8	156.3
HJ96AS-1	6	113.8	347.9	650.9	1250.0
H	7	21.1	44.3	99.2	312.5
B	7	43.1	83.8	121.9	156.3
MJ99-132	8	101.3	326.1	627.9	1250.0
HJ00-4	9	105.5	430.4	946.6	1250.0
24-h recovery					
Isolate	Subtype	IC_50_ (ppm)	IC_90_ (ppm)	IC_99_ (ppm)	MLC (ppm)
NUH9	1	118.7	344.5	1101.0	2500.0
HJ96-1	2	69.7	206.7	501.4	1250.0
HJ96A-26	3	77.0	129.7	173.6	312.5
S1	4	99.9	252.2	692.6	1250.0
WR1	4	83.9	207.0	554.9	1250.0
SY94-3	5	35.3	357.5	1310.0	2500.0
HJ96AS-1	6	307.1	1243.0	2724.0	5000.0
H	7	30.4	54.6	103.3	312.5
B	7	59.9	84.1	172.8	312.5
MJ99-132	8	357.5	1438.0	3138.0	5000.0
HJ00-4	9	591.6	1793.0	3338.0	5000.0

IC, inhibitory concentration; MLC, minimum lethal concentration. Axenic cultures in blue; xenic cultures in red

Recovery after 24 h of incubation without hydrogen peroxide treatment exhibited higher IC values than that of those corresponding to the 24-h treatment assay, suggesting that resistance forms (cysts) are also developed during hydrogen peroxide treatment (Fig. 2). Hence, the effective hydrogen peroxide concentrations are even higher than those previously indicated (Table 3, 24-h recovery). All *Blastocystis* isolates showed resistance to hydrogen peroxide, with concentrations ranging from 103 ppm to 3,338 ppm for 24 h to completely eliminate any chance of recovery (Table 3, 24-h recovery). Herein, both ST8 and ST9 showed the highest resistance to hydrogen peroxide.

### Fluorescence live-cell imaging

**Fig. 3:**
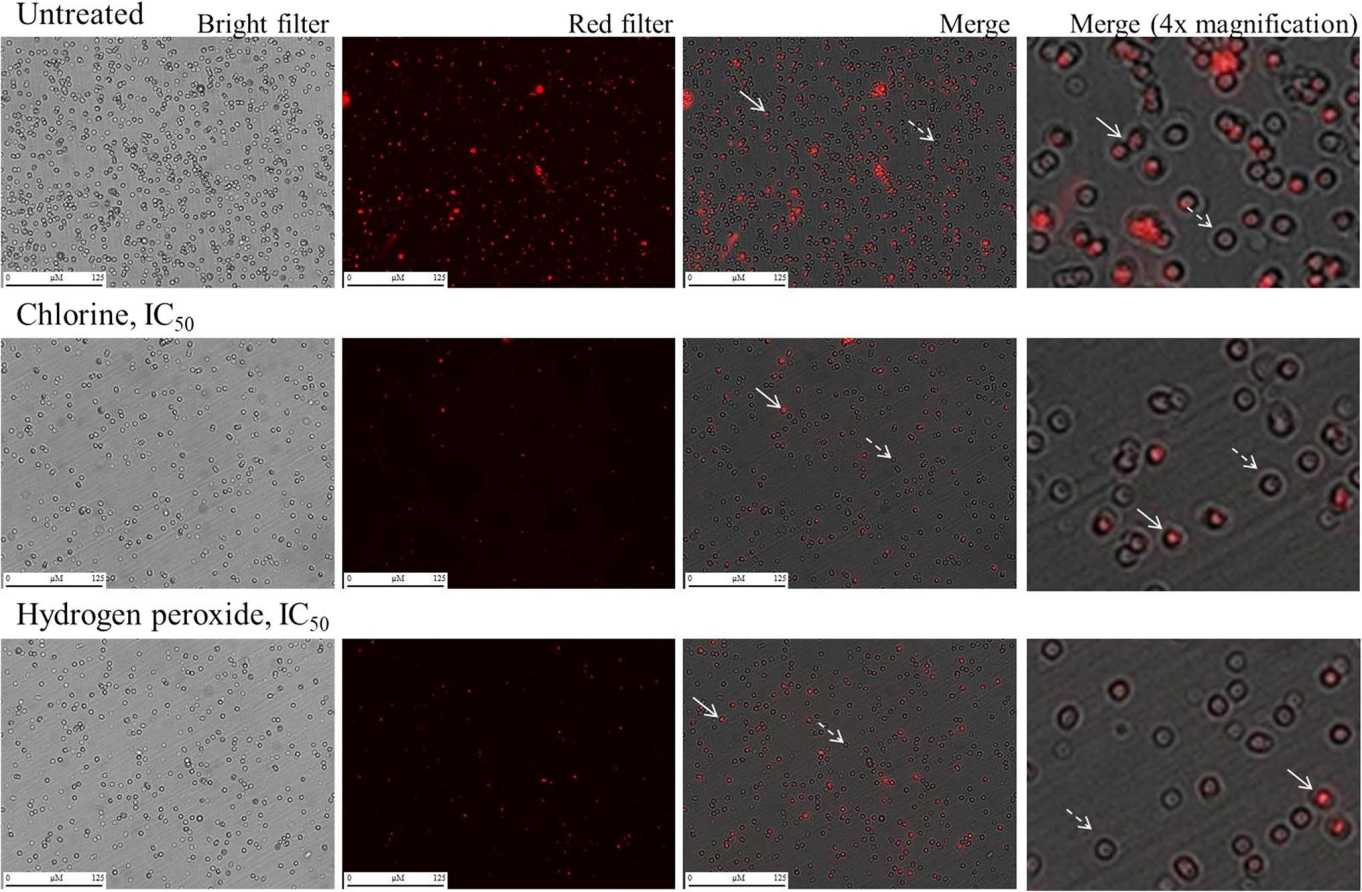
Representative microscopic images of *Blastocystis* ST4 S1 untreated (control) and treated at IC_50_ concentrations of chlorine and hydrogen peroxide for 24 h and stained with MitoTracker.™ Red CMXRos. Arrows point to active cells; dashed arrows point to non-active cells (dead cells/cysts)

To visualize the effect of these treatments at the cellular level, we randomly generated and collected microscopic images of *Blastocystis* treated at IC_50_ concentrations of chlorine and hydrogen peroxide for 24 h (Fig. 3). Live *Blastocystis* cells were stained with MitoTrackerTM Red CMXRos. Images showed that both the number of total cells and the percentage of live (stained) cells were lower in the treated cultures than in the control (untreated) cultures for all isolates tested.

## Discussion

Water is a common vehicle for transmission of many pathogenic and nonpathogenic organisms, including *Blastocystis* (Jinatham et al. 2021, 2022). Chlorine is one of the most widely used reagents for water disinfection. Concentrations of 0.2–1.0 ppm (0.2–1.0 mg/L) of chlorine are effective for eradicating most pathogens, while levels up to 5.0 ppm are considered safe in drinking water (Centers for Disease Control and Prevention 2020). In instances of over chlorination (8.0–10.0 ppm), the World Health Organization (WHO) recommends implementation of dechlorination treatment to make it suitable for human consumption (Zaki et al. 1996). In this respect, countries treat drinking water with chlorine up to 0.2–5.0 ppm, depending on local drinking water regulations (Karim et al. 2020). In swimming pools, chlorine levels are regulated to be within the range of 0.3–5.0 ppm in several countries (Yang et al. 2018). However, health institutions and agencies, including the WHO and the Centers for Disease Control and Prevention (CDC), report that chlorination is not as effective against protozoa and fungi (WHO. World Health Organization 1982; Centers for Disease Control and Prevention 2022). Thus, higher concentrations of chlorine than those considered safe for human consumption should be used in order to eradicate them. In this regard, it would be interesting to investigate whether the approved levels of chlorination affect *Blastocystis* viability.

Low concentrations of chlorine (< 5 ppm) have a biocidal effect on a number of bacteria – 25 ppm on *Mycoplasma*, 100 ppm on *Bacillus atrophaeus* spores, 200 ppm on a number of viruses, and 500 ppm on *Candida* spp.; higher concentrations are required to eliminate *Mycobacterium tuberculosis* (1,000 ppm) or inactivate *Clostridium difficile* spores (5,000 ppm) (Centers for Disease Control and Prevention, National Center for Emerging and Zoonotic Infectious Diseases (NCEZID) 2016). In this study, we demonstrated that all *Blastocystis* isolates included were highly resistant to chlorine, requiring concentrations ranging from 175 ppm to higher than 1,800 ppm to eliminate any chance of recovery. Among the nine *Blastocystis* subtypes investigated herein, ST1 (strain NUH9) and ST7 (strain B) were the most resistant to chlorine during treatment and recovery. Notably, ST1 is among the most prevalent and widely distributed subtype in humans globally, while ST7 is common in poultry and quite common in some human populations (Alfellani et al. 2013a). Previous findings suggesting water as a prominent transmission route of *Blastocystis* along with the chlorine resistance identified in the present study might help explain how these two subtypes persist in the environment. Moreover, among the rest of the subtypes, all, except ST6, show elevated resistance post recovery suggesting the presence of a resistance mechanism against chlorine in the genus. It is worth noting that most of the cultures are xenic, and while the values could be associated with the overall culture microbiome, we have not observed any consistent differences between xenic versus axenic subtypes.

In parallel, hydrogen peroxide has biocidal effect against a wide range of viruses, bacteria, protozoa, and fungi. Hydrogen peroxide at 5,000 ppm has virucidal and fungicidal effects after 5 min of exposure and a broad bactericidal effect after 60 min. A concentration of 30,000 ppm eliminates *Bacillus* spp. spores after 150 min of exposure. However, the same concentration is ineffective against vancomycin-resistant enterococci and *Acanthamoeba* cysts after 120 min of exposure (Centers for Disease Control and Prevention, National Center for Emerging and Zoonotic Infectious Diseases (NCEZID) 2016). In this study, we demonstrated that all *Blastocystis* isolates studied were slightly resistant to hydrogen peroxide, requiring concentrations ranging from 103.3 ppm to 3,338.0 ppm for 24 h to eliminate any chance of recovery. These results suggest that hydrogen peroxide at concentrations usually used for disinfection against many other microorganisms is more than adequate for the effective treatment of surfaces, tools, or fabrics against *Blastocystis*. At the level of subtypes, ST9, ST6, and ST1 showed the highest resistance to the reagent. In our previous study, using hydrogen peroxide exposure in ST1 (strain NandII), we showed similar findings along with upregulation of genes related to oxygen stress (Tsaousis et al. 2012). At the genomic level resistance to oxygen stress has been predicted in silico in various subtypes (Denoeud et al. 2011; Eme et al. 2017; Gentekaki et al. 2017).

Future studies should focus on investigating the molecular mechanisms of additional subtypes and strains within subtypes in developing resistance to both chlorine and hydrogen peroxide but also on the strategies that *Blastocystis* cells have evolved to initiate both encystation and excystation and how these do affect the transmission of the organism. Moreover, the use of additional contact times and incubation in different temperatures (e.g., ambient temperature) should also be considered in the future. One limitation of the study herein is the lack of information regarding the amount of cyst forms in each condition, but this is due to the unavailability of markers to confirm this stage.

Collectively, the biochemical and cell biological results herein suggest that other water treatment processes, either chemical or physical, should be applied to eliminate *Blastocystis* in water. For instance, prechlorination treatment stages such as sedimentation, coagulation, flocculation, and filtration should be used in the water disinfection procedure. In rural areas, where it is often not possible to include these necessary treatment stages, *Blastocystis* remains in the water maintaining transmission cycles.

## Supplementary Information

Below is the link to the electronic supplementary material.

Supplementary file1 (DOCX 9347 KB)

## Data Availability

The datasets generated during and/or analyzed during the current study are available from the corresponding author on reasonable request.
