# Supplementary material for: Resistance of Blastocystis to chlorine and hydrogen peroxide
Source: Parasitol Res. 2022 Nov 15;122(1):167–76. doi: 10.1007/s00436-022-07713-2 (PMC9816239; doi:10.1007/s00436-022-07713-2)
Supplement: Supplementary file 1 — Supplementary file1 (DOCX 9347 KB) [file 436_2022_7713_MOESM1_ESM.docx]

**
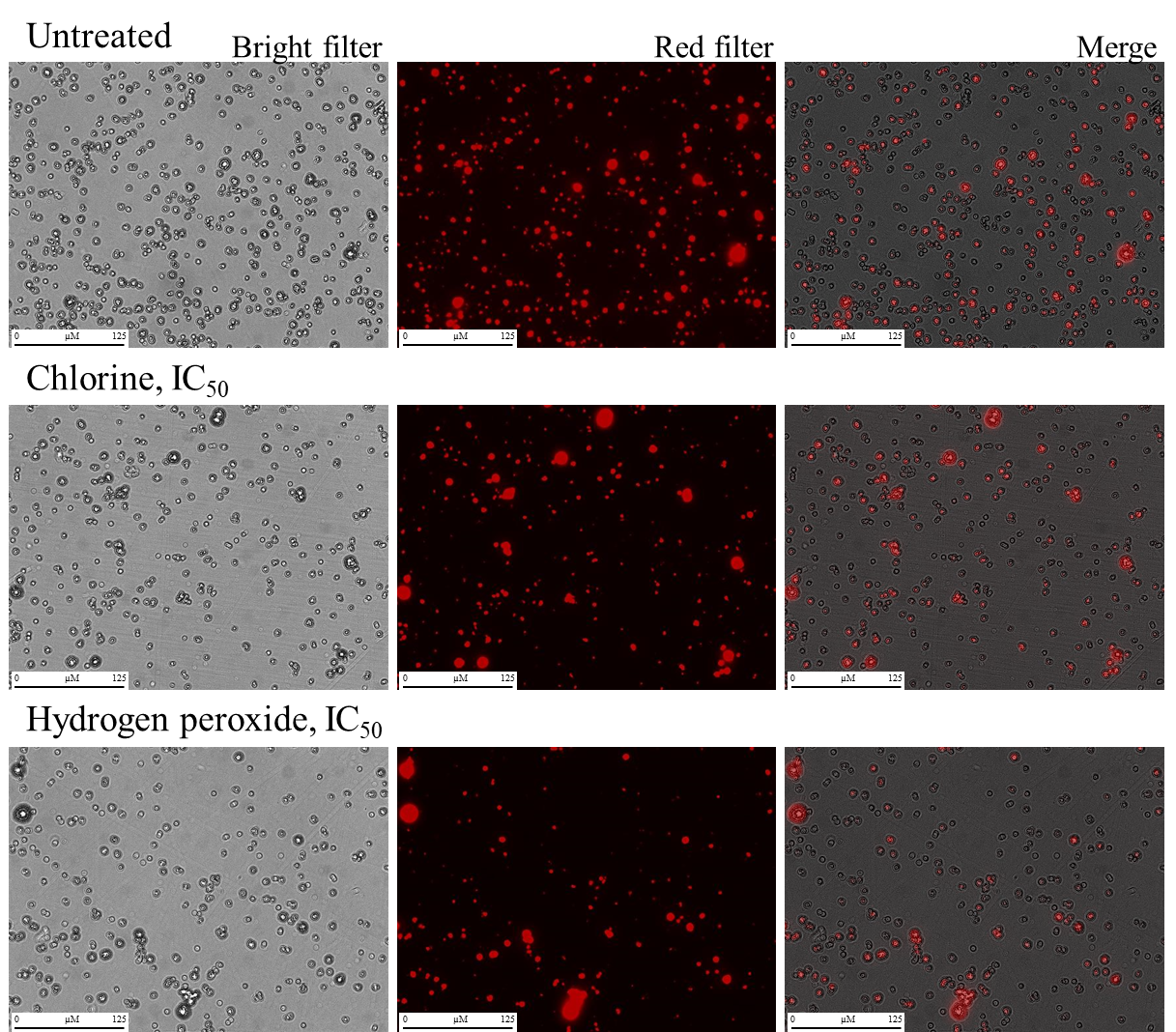
**

**Figure S1**. Representative microscopic images of *Blastocystis* ST4 WR1 untreated (control) and treated at IC_50_ concentrations of chlorine and hydrogen peroxide for 24 h, and stained with MitoTrackerTM Red CMXRos.

**
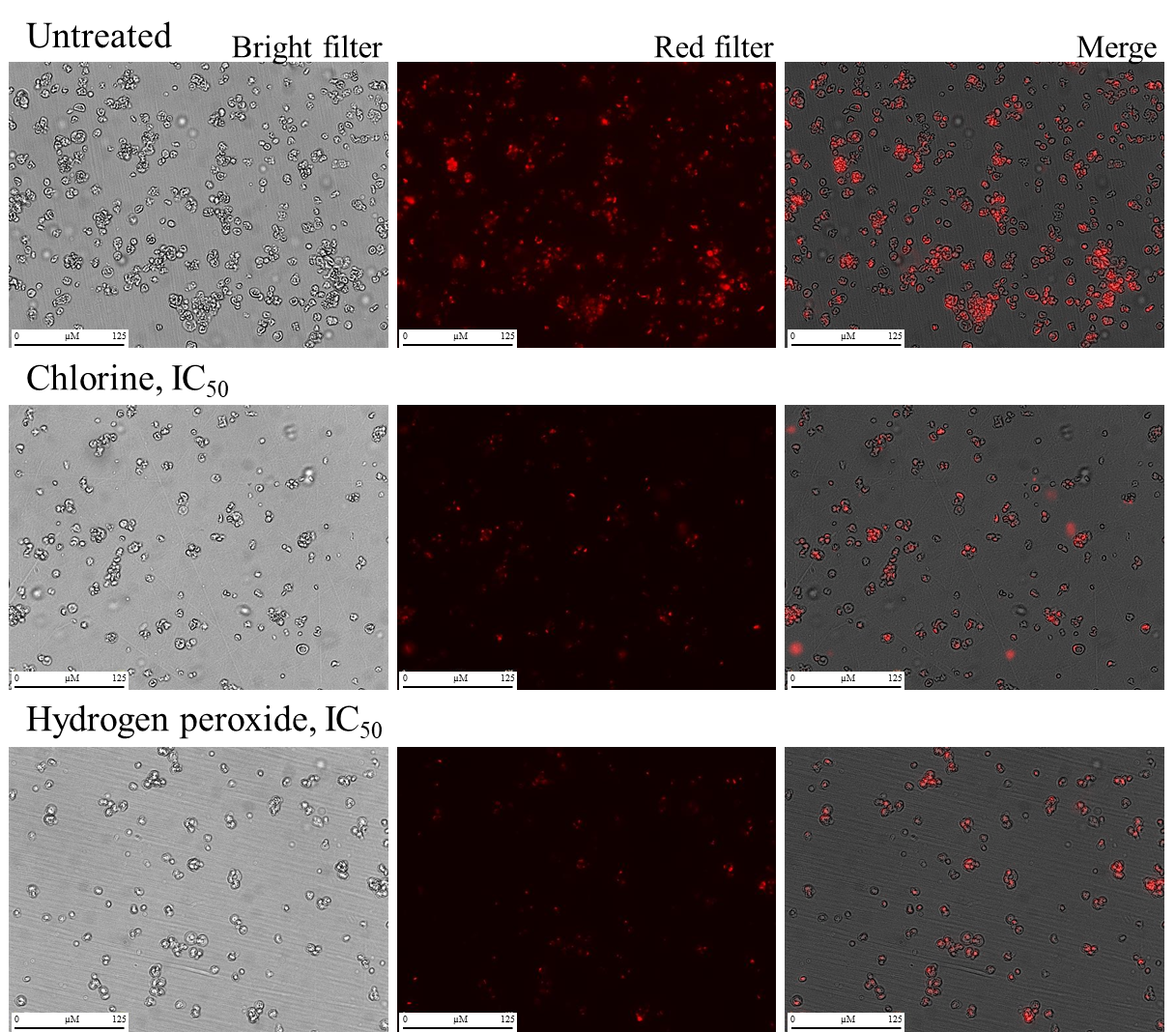
**

**Figure S2**. Representative microscopic images of *Blastocystis* ST7 H untreated (control) and treated at IC_50_ concentrations of chlorine and hydrogen peroxide for 24 h, and stained with MitoTrackerTM Red CMXRos.

**
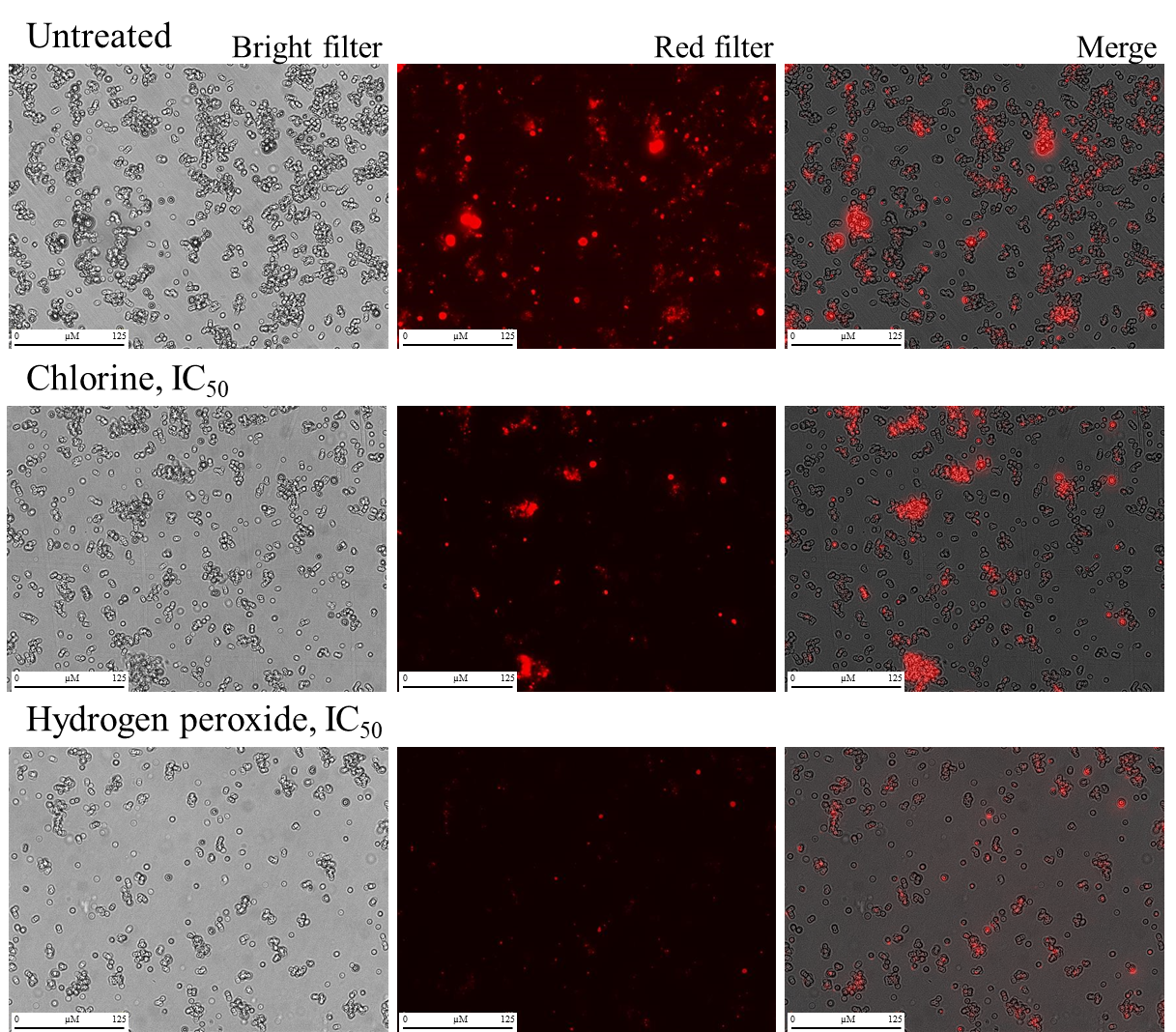
**

**Figure S3**. Representative microscopic images of *Blastocystis* ST7 B untreated (control) and treated at IC_50_ concentrations of chlorine and hydrogen peroxide for 24 h, and stained with MitoTrackerTM Red CMXRos.

Table S1: Number of *Blastocystis* cells obtained through the JuLi system software (see Figure S4) that were used to calculate the cell viability.

| **Sample** | **Count** | **Total cells vs untreated** | **Active cells vs untreated** |
| --- | --- | --- | --- |
| **Untreated** | | | |
| Total cells (Bright) | 874 | 100% | - |
| Active cells (Red) | 723 (83%) | - | 100% |
| **Chlorine, IC_50_** | | | |
| Total cells (Bright) | 327 | 37% | - |
| Active cells (Red) | 211 (64%) | - | 29% |
| **Hydrogen peroxide, IC_50_** | | | |
| Total cells (Bright) | 308 | 35% | - |
| Active cells (Red) | 213 (69%) | - | 29% |

In brackets, percentage of active cells

**Figure S4**. Representative microscopic images of *Blastocystis* ST4 B untreated (control) and treated at IC_50_ concentrations of chlorine and hydrogen peroxide for 24 h, and stained with MitoTrackerTM Red CMXRos. Arrows show cells that have been selected as inactive (e.g. potential cysts).
